# Women suffering from overactive bladder syndrome exhibit a higher urethral viral abundance compared to healthy controls: a pilot study

**DOI:** 10.1038/s41598-025-98780-9

**Published:** 2025-06-03

**Authors:** Marianne Koch, Sara Lado, Barbara Bodner-Adler, Greta Carlin, Cátia Pacífico, Caroline Bauer, Rufus Cartwright, David Seki, Christoph Steininger, Athanasios Makristathis, Wolfgang Umek

**Affiliations:** 1 Department of Obstetrics and Gynecology, Medical University of Vienna, Spitalgasse 23, Vienna, 1090 Austria; 2 Department of Internal Medicine I, Division of Infectious Diseases and Tropical Medicine, Medical University of Vienna, Vienna, Austria; 3 Karl-Landsteiner Institute for Microbiome Research, Medical University of Vienna, Vienna, 1090 Austria; 4 Unit of Food Hygiene and Technology, Institute of Food Safety, Food Technology and Veterinary Public Health, University of Veterinary Medicine Vienna, Vienna, Austria; 5 Department of Obstetrics and Gynecology, Chelsea & Westminster NHS Foundation Trust, London, UK; 6 Division of Clinical Microbiology, Department of Laboratory Medicine, Medical University of Vienna, Vienna, Austria; 7 Joint Microbiome Facility of the Medical University of Vienna and the University of Vienna, Vienna, Austria; 8 Centre for Microbiology and Environmental Systems Science, Department of Microbiology and Ecosystem Science, Division of Microbial Ecology, University of Vienna, Vienna, Austria; 9 Karl-Landsteiner Institute for special gynecology and obstetrics, Vienna, Austria

**Keywords:** Urogenital diseases, Urinary incontinence, Viral reservoirs

## Abstract

The interactions between the human bacterial microbiome and essential bodily functions are well established for organ systems such as the oral cavity, gut, and female reproductive tract. However, the urinary microbiome, particularly its viral component, remains largely unexplored. Emerging evidence suggests that the urinary microbiome may play a significant role in the development of overactive bladder syndrome (OAB). This study aims to fill this knowledge gap by investigating the potential link between the urethral virome and female overactive bladder syndrome, and by aligning these findings with the bacterial microbiome. Prospective pilot study including 15 patients with overactive bladder syndrome and five controls. Current urinary tract infection and antibiotic therapy within the last two months were ruled out and controls were matched to cases by age and body mass index. Urethral swabs **(**Copan eSwab^®^ urethra) were taken from each participant at one single time point. Subsequent viral isolation, purification, and enrichment were conducted using the ViPEP method. Next-generation sequencing was performed on pooled samples, followed by bioinformatic analysis to identify and classify viral contigs. Phylogenetic analysis was conducted to assess genetic relationships among identified viral sequences. The bacterial microbiome was analyzed by sequencing of the variable V3-4 region of the eubacterial 16 S rDNA gene on the Illumina MiSeq platform. We identified twenty-one viruses and bacteriophages only in pooled urethral swab samples of the OAB group, but no valid detections were retained in the control group after analysis. The most abundant human virus in urethral swab samples was human papilloma virus, whereas the most abundant bacteriophages belong to the family of Siphoviridae. In the bacterial microbiome analysis, we identified statistically higher levels of *Veillonella* and *Bacteroides* in OAB samples. Results of this pilot study suggest a difference in the urethral virome between women with OAB and healthy controls. When looking deeper into the detected virus families and species, we might postulate a unique microbial pattern of OAB patients. This pattern suggests an interplay of immunosuppression, autoimmune processes and a larger diversity of bacterial and viral microbes. Current evidence strongly suggests a disturbance of the healthy microbiome of the urogenital tract in patients with OAB, leading to subclinical chronic inflammation and thus typical OAB symptoms. Further research should focus on interventions aimed at restoring a healthy microbiome in OAB patients to mitigate inflammation and improve symptom control.

**Supplementary Information:**

The online version contains supplementary material available at 10.1038/s41598-025-98780-9.

## Introduction

### Background and rationale

In recent years, studies on the human microbiome have greatly improved our understanding of the human body and the development of diseases in many areas^1^. The human microbiome comprises the entirety of bacteria, viruses, and fungi that colonize the human body and which do not primarily imply pathogenicity. Whereas the human bacterial microbiome is well established in some areas, especially the human gut, less is known about the human viral microbiome (virome) and urinary tract microbiome and its relation to lower urinary tract symptoms^1,2^.

Overactive bladder syndrome (OAB) is characterized by urinary urgency, frequent toileting, nocturia and with or without urinary incontinence^3^. About 13% of women in the general population are affected by OAB, with prevalence rates increasing to 20% over the age of 70 years and reaching a maximum of 48% in obese women^4,5^. Women with OAB report social isolation, loss of confidence, depression, limitation of daily activities, disturbance of sexual health and a general reduction of quality of life^6^. Despite the high prevalence of OAB and large socio-economic implications, the etiology is yet largely unknown and it is currently only assumed that neuronal, myogenic and inflammatory factors each play their role in the development of OAB^7–9^. The diagnosis of OAB is primarily clinical, based on patient-reported symptoms, and is considered a diagnosis of exclusion, meaning other potential causes must be ruled out first^10–12^. Initial evaluation typically involves a targeted history and examination of the urogenital system to assess the burden of the disease on the patient^10^. Guidelines suggest that invasive testing is generally not required for uncomplicated cases^13,14^. The first-line treatment for OAB includes lifestyle modifications, bladder training, pelvic floor exercises, and dietary changes^10^. If behavioral therapies are insufficient, medications such as antimuscarinic agents and beta-3 adrenergic agonists are commonly prescribed. For patients who do not respond to first-line treatments, options such as intradetrusor injections of onabotulinumtoxinA, sacral neuromodulation, and percutaneous tibial nerve stimulation are available^12,15^. Previous microbiome studies reported differences in the bacterial microbiome of patients with OAB when compared to controls, including a larger diversity of bacteria and lesser abundance of *Lactobacillus* species in OAB patients^16–19^. Whereas the urinary bacterial microbiome has received some research attention, less is known about the urinary viral microbiome. Some studies have identified a shedding of eukaryotic viruses of the *Polyomaviridae* family in urine of asymptomatic humans^20,21^. A single study has postulated an association between the human urine viral microbiome, and acute urinary tract infection^17^. Comparing the urine of patients with acute urinary tract infection to healthy ones, sequencing identified mostly bacteriophages, as well as eukaryotic viruses, and in almost all samples, reads from human papillomaviruses^22^. Whereas the urinary bacterial microbiome seems to differ between OAB patients and healthy controls, there is yet no data available on the viral microbiome in the context of OAB. Previous publications generally suggest an essential role of human gut viral phage communities in controlling bacterial communities. The gut viral microbiome can alter the bacterial microbiome by infection of specific bacterial populations. Thus, the human viral microbiome including bacteriophages is assumed to influence microbiotal homeostasis or dysbiosis, and therefore to possibly contribute to a variety of individual conditions, such as autoimmune diseases or chronic infectious diseases^23^.

### Objectives

The primary objective of this study was to analyze the composition of the urethral virome, specifically focusing on potential differences between women diagnosed with overactive bladder syndrome and those without such symptoms, serving as controls. In a secondary approach we aimed to align the detected the urethral virome with bacterial microbiome. Ultimately, the overarching goal of this study was to contribute to a deeper understanding of the complex interplay between microbial communities and the manifestation of OAB symptoms, with the ultimate aim of improving patient care and management of this prevalent and burdensome condition.

## Methods

### Study design

We conducted a pilot study comparing the urethral virome of 15 patients with overactive bladder syndrome and five healthy controls.

### Setting and participants

Participants were recruited at the urogynecology outpatient department (patients with overactive bladder syndrome) and the general gynecology department (controls) of the Department of Obstetrics and Gynecology, Medical University of Vienna. This research project was approved by the Ethics Committee of the Medical University of Vienna (2160/2019) and written informed consent was obtained from all participants. We confirm that all experiments were performed in accordance with relevant guidelines and regulations and all study procedures were in accordance with the Declaration of Helsinki.

We defined the following exclusion criteria: any neurological conditions that may affect the lower urinary tract, diagnosis of interstitial cystitis or painful bladder syndrome, acute urinary tract infection (confirmed by dipstick urine test) or antibiotic therapy during the past 8 weeks. Controls were matched by age (+/- 5 years) and Body Mass Index (18.5–24.9 kg/m^2^ normal weight; 25–29.9 kg/m^2^ overweight; 30–34.9 kg/m^2^ obesity grade I). All participants completed the ICIQ- OAB questionnaire (International Consultation of Incontinence Questionnaire for overactive bladder syndrome). Patients were eligible for the control group only if they did not report any symptoms of overactive bladder syndrome and if they had a score of < 5 at the ICIQ questionnaire. The diagnosis of overactive bladder was made if patients subjectively reported symptoms of urgency and frequency with or without urinary incontinence, and if this was consistent with their 3-day bladder diary, the ICIQ-questionnaire results and bladder sensation during cystometry results (standardized filling of the bladder with a water filling system (saline at room temperature; approximately 23 °C) at a supraphysiologic filling rate of 50 ml/min via a transurethral catheter; subsequent recording of „first bladder sensation“, “first desire to void“ and “strong desire to void”). Filling cystometry was performed to evaluate the bladder filling capacity to rule out other conditions such as polydipsia with overflow bladder. A urethral swab was collected from all participants at a second follow-up visit using the Copan eSwab^®^ Urethra swab. Patients were not catheterized before swab retrieval. Swabs were then diluted in PBS buffer (phosphate- buffered saline) pH7 to conserve intact virions, as described previously^24^, and stored at − 80°C until further processing.

### Study size

Since this was a pilot study, we defined a sample size of 20 (15 cases; 5 controls) for primary analysis.

### Laboratory analysis of viral Microbiome

In a next step the samples were pooled into four pools of five samples each - three pools of positive patients and one pool of healthy patients. For the pools, 100 µl per sample was processed together in a 1:5 dilution with PBS, as PBS stabilizes the viral particles.

### Virus purification and enrichment protocol (ViPEP)

After sample preparation, viral material was targeted following VIPEP (viral isolation, purification and enrichment procedure)^24^. With this method, we could efficiently purify viral particles from low volumes of clinical specimens. In short, virus particles were enriched by a nine-step procedure that includes initial resuspension of the homogenates in DPBS buffer pH7 (Dulbecco’s PBS, no calcium, no magnesium, Thermo Fisher Scientific), two centrifugation steps for 5 min at 2500× g and 15 min at 4800× g, filtration through a 0.45 µM syringe filter, ultrafiltration using 50 kDa molecular weight cut-off filtration units (Amicon Ultra-15 50 K, Merck Millipore, Cork, Ireland), DNase I digestion (Qiagen), DNA and RNA preparation using a QIAamp UCP Micro Kit (Qiagen), blocking of ribosomal RNA sequences using a set of 5 specific oligonucleotides, cDNA synthesis using nonribosomal hexanucleotides together with the Superscript IV enzyme (Thermo Fisher Scientific). A final amplification of the total nucleic acids was performed using a Repli-g kit (Qiagen, Hilden, Germany) to increase the DNA yield. Genomic DNA was quantified by using Qubit dsDNA High Sensitivity Kit on a Qubit fluorometer (Thermo Fisher Scientific, Santa Clara, California, USA).

### Library Preparation and next generation sequencing for viral Microbiome analysis

Individual samples were prepared using the NEBNext^®^ Ultra™ II FS DNA Library Prep Kit for Illumina following the protocol for low sample inputs (< 100ng). The fragment sizes and quality of the individually prepared libraries was checked on an Agilent High Sensitivity D1000 Tape, using the Agilent Tape Station 4150 device. Following fluorometric DNA quantification with Qubit 4.0 (Thermo Fisher Scientific), the barcoded libraries were equimolarly pooled. The final insert size and concentration of the pool was determined (Agilent High Sensitivity D1000 Tape and Qubit™) and prepared for MiSeq sequencing using the MiSeq Reagent Kit V2 (Illumina). The library was sequenced on a 2 × 150 bp V2 MiSeq flowcell (Illumina) with 300 cycles.

### Laboratory analysis of bacterial Microbiome

DNA extraction from urethral swab samples was performed by MasterPure Complete DNA & RNA Purification Kit (Lucigen). The variable V3-4 region of the eubacterial 16 S rDNA gene was amplified. Sample library preparation was performed according to the Illumina protocol (Illumina, San Diego, USA) followed by sequence analysis on the Illumina MiSeq platform.

### Bioinformatic analysis

#### Viral Microbiome

The quality of raw sequencing reads was first manually inspected using FastQC v0.11.9 and MultiQC v1.13^25,26^. Illumina adaptor sequences and reads with low quality scores (below 20), a length below 30 nt and N´s at the end of the reads were removed using cutadapt v4.1^27^. Host contaminant (human version GRCh38) was removed from the trimmed and filtered sequencing reads using Bowtie2 v2.4.5^28^, keeping only unmapped reads. Negative controls were *de novo* assembled using MEGAHIT v1.2.9^29^ with default parameters and used as indexes for Bowtie2. Only reads that did not map to the negative controls were kept for downstream analysis. We also used seqtk v1.3-r106^30^ to subsample an overclustered sample (pool one) to 450,000 read pairs. Metagenome assemblies were conducted using SPAdes v3.15.2^31^ with the metagenome option. For the SPAdes assembly only the representative subsample was analyzed (the original was too heavy to analyze). Contigs were deduplicated using CD-HIT-EST v.4.8.1^32^ at a minimum 95% identity and 95% overlap. Only contigs longer than 200 nt passed to the next step. Taxonomic classification of deduplicated contigs against a viral database (2022-03-29) were performed using Kaiju v1.6.2^25^ with 75 minimum match score. Contamination from an environmental project was manually excluded from the results, by comparing both project results, which allowed for straightforward detection and subsequent elimination.

#### Bacterial Microbiome

Microbial reads were quality-filtered and demultiplexed, followed by amplicon sequencing variants (ASV) inference with DADA2 v1.20^33^ applying the recommended workflow^34^ FASTQ reads 1 and 2 were trimmed to 230 nucleotides, with allowed expected errors of 4 and 6, respectively. ASV sequences were subsequently classified using DADA2 and the SILVA database (SSU Ref NR 99 release 138.1)^35^ with a confidence threshold of 0.5. ASV without classification or classified as mitochondria, as well as well-known buffer contaminants, were removed^36^. 16 S rRNA gene counts were normalized via rarefying to even minimal sampling depth (5000 reads per sample). Subsequently, a filter was applied to exclude ASVs that occurred below 1% relative abundance. Downstream, Permanova and analysis of alpha and beta diversity were performed in R version 4.0 and the R package vegan^37^. T-tests and calculation of effect sizes (Cohen’s d) were performed via rstatix^38^, and p-values were adjusted using the method of false discovery rate (FDR). Data was visualized via R version 4.0 and R package ggplot2 version 3.3.342.

### Phylogenetic tree of HPV types

Sequence alignments were performed with BioEdit^39^ using nucleotide sequences, by using our detected HPV contigs and aligning them to reference sequences from Genbank from different papillomavirus families. The nucleotide sequences were then translated into an amino acid sequences and phylogenetic trees were performed using MEGA11^40^. Each phylogenetic tree was assembled using the Maximum Likelihood method and a Jones-Taylor-Thornton model^41^. The test of phylogeny was performed with the Bootstrap method with 10,000 replicates. Branches that belong to partitions that have been replicated in fewer than 50% of bootstrap replicates are collapsed. The proportion of replicate trees where the relevant taxa grouped together in the bootstrap test (10000 repetitions) is displayed next to the branches.

## Results

Background characteristics of patients and controls are summarized in Table 1.

**Table 1:** Patient characteristics

	All (*n* = 20)	Cases (*n* = 15)	Controls (*n* = 5)
*Median (IQR)*			
Age (years)	61 (56–70)	63 (56–72)	60 (56–61)
BMI (kg/m^2^)	27.49 (25–29)	28.28 (26–30)	21.36 (21–27)
Vaginal deliveries (n)	1 (0–2)	1 (0–2)	0 (0–0)
POP-Q stage (1–3)	2 (2–2)	2 (2–2)	NA
Diuria (n)	7.75 (4–10)	9.5 (4–11)	3.5 (4–8)
Nocturia (n)	2 (1–3)	2 (2–3)	1 (1–1)
ICIQ sum score	20.5 (8–28)	26 (16–38)	0 (0–2)
Cups of coffee/day (n)	2 (1–3)	2 (1–3)	2 (1–2)
Liquid intake/day (litre)	1.5 (2–2)	1.5 (2–2)	1.5 (2–3)
Oxford scale	2 (2–3)	2 (2–3)	NA
*Results of cystometry (ml)*			
First bladder feeling	120 (70–145)	120 (70–145)	NA
First urge	205 (142–245)	205 (142–245)	NA
Imperative urge	240 (185–250)	240 (185–250)	NA
Bladder filling stopped	300 (300–300)	300 (300–300)	NA
*N (%)*			
Polypharmacy*			
Yes	2 (10%)	2 (13.33%)	0 (0%)
No	18 (90%)	13 (86.67%)	5 (100%)
Comorbidities**			
Yes	16 (80%)	13 (86.67%)	3 (60%)
No	4 (20%)	2 (13.33%)	2 (40%)
*Fecal Incontinence*			
Yes	1 (5%)	1 (6.67%)	0 (0%)
No	19 (95%)	14 (93.33%)	5 (100%)
*Pelvic organ prolapse*			
Yes	8 (40%)	8 (53.33%)	0 (0%)
No	12 (60%)	7 (46.67%)	5 (100%)
*OAB wet*			
Yes	14 (70%)	14 (93.33%)	0 (0%)
No	6 (30%)	1 (6.67%)	5 (100%)
*Mixed urinary incontinence*			
Yes	4 (20%)	4 (26.67%)	0 (0%)
No	16 (80%)	11 (73.33%)	5 (100%)
*Nicotine*			
Yes	2 (11.11%)	2 (15.38%)	0 (0%)
No	16 (88.89%)	11 (84.62%)	5 (100%)
*Alcohol consumption*			
Never	9 (50%)	6 (46.15%)	3 (60%)
Regularly	4 (22.22%)	3 (23.08%)	1 (20%)
Seldomly	4 (22.22%)	3 (23.08%)	1 (20%)
Sometimes	1 (5.56%)	1 (7.69%)	0 (0%)

*Polypharmacy including: hormonal replacement therapy for menopause, thyroid supplementation, antihypertensives, anticholinergics, vitamin D supplementation, acetylsalicylic acid, antidepressants, metformin, cholesterol lowering drugs, pantoprazole, hydrocortone **Comorbidities include (case group): hypothyroidism, hypertension, Intestinal and endometrial polyps, Lichen sclerosus, Depression, chronic pelvic pain, liver cirrhosis, diabetes mellitus, gastritis, multiple myeloma, uterine leiomyoma, hypercholesterinemia; Comorbidities include (control group): Morbus Addison, hypertension.

### Sequencing viral data

After processing raw data and eliminating reads that aligned to the host and to the negative control (Supplementary Table 1), we used SPAdes assembler (open source tool for de novo sequencing^31^ for the mapping of reads into contigs (Supplementary Table 2). After filtering the contigs with minimum size of 200 bp (Supplementary Table 3), we ran the software Kaiju for taxonomic classification.

### Preliminary results of Virome analysis

According to Kaiju, the percentage of viral contigs of all SPAdes assembled contigs higher than 200 bp was minimal: pool one 1%, pool two 0.7%, pool three 0.8% and pool four 0.6%. In this primary analysis, the BeAn 58,058 virus showed the highest abundance in all pools. An additional blast of different contig sequences using BLAST ^®^ tools however identified these hits as human reads (Supplementary Tables 4 and Table 5) or different human retroviruses, or BeAn58058. With our Kaiju results, a mean abundance of 70% (values between 45 and 86%) was detected. Those values are close to other studies, where they detected the BeAn 58,058 virus from human samples^42,43^. To take a closer look at BeAn 58,058, several contigs of all pools were aligned to the reference genome of the BeAn 58,058 virus (NC_032111) and the reference genome of the HIV isolate (MZ766785) acquired from the BLAST ^®^ analysis (Supplementary Fig. 1). Most contigs align to the viruses in a short highly conserved region of a length of approximately 200 bp(~ 8300–8600 bp), overlapping partly the Kelch repeat and BTB domain-containing protein A55 (HIV isolate), and as seen in other studies a simple repeat region that falls into the class of Alu elements (BeAn 58058). As this sequence aligns to different viruses and the BLAST ^®^ results show that the contigs can be of human origin (Supplementary Table 4), the occurrence of the BeAn 58,058 virus in human samples is questioned. Hence, it seems plausible that in fact human DNA was detected rather than BeAn58058 virus, since the sequence is within a highly repetitive region.

Further Kaiju human virus predictions were blasted using BLAST ^®^ tools. For the Human papillomavirus type 53 contig identified with the SPADES assembler in pool one, BLAST ^®^suggested the closest virus to be HPV type 56 instead of 53 (Supplementary Table 6). This was additionally controlled with an alignment on the capsid protein L1 region, of the predicted HPV contig 1250 with the reference accession number “OP971017.1” of HPV type 56, with a fragment of 272 bp. After transforming the alignment to aminoacid sequences, a phylogenetic tree was created to confirm this (maximum likelihood tree; Supplementary Fig. 2). The contig 1250 aligns with high similarity to the reference genome of HPV 56 and clearly shares a most common recent ancestor with the HPV 56 reference genomes. The BLAST ^®^ result for the contigs 1429 and 1658 of pool 1, which were identified with Kaiju to belong the group of Alphapapillomavirus 3, showed a closer hit for human papilloma virus type 87 (KU298942.1; Supplementary Table 6). The same happened to the Macaca mulatta papillomavirus 6 contig, from pool 1. As the three contigs are in different regions of the human papilloma virus type 87 genome, a consensus sequence from the three contigs was created to do a phylogenetic tree. For that, we added to the nucleotide alignment several reference genomes into a 780 bp alignment, and translated the alignment into aminoacids. The tree is showing HPV type 87 to share a most common recent ancestor to our contigs, compared to other HPV types (Supplementary Fig. 3).

### Main results of Virome analysis

We identified viruses (which infect and replicate with humans) and phages (which infect and replicate with bacteria) only in pooled urethral swab samples of the OAB group, but no valid detections were retained in the control group after analysis. The most abundant human virus in urethral swab samples was Human Papilloma Virus, whereas the most abundant bacteriophages belong to the family of Siphoviridae (Table 2).

Table 2 shows the results of the viral metagenomic analysis of urethral swab samples from patients with overactive bladder syndrome (OAB) and healthy controls. The table lists various viral contigs that were identified in the samples, along with their NCBI taxonomy classification, including family, species, and host. The table also shows the number of contigs present per sample for each virus in both the OAB and control groups.

**Table 2:** Metagenomic Virome richness.

NCBI_tax	Family	Species	Host	Positive OAB			Negative
				1 (*n* = 5)	2 (*n* = 5)	3 (*n* = 5)	4 (*n* = 5)
120,381	Papillomaviridae	Human papillomavirus type 87	HUMAN	3	0	0	0
10,596	Papillomaviridae	Human papillomavirus type 56	HUMAN	1	0	0	0
687,358	Anelloviridae	Torque Teno Virus 19	HUMAN	1	0	0	0
687,368	Anelloviridae	Torque Teno Virus 29	HUMAN	0	1	0	0
166,122	Retroviridae	Human endogenous retrovirus K113	HUMAN	1	0	1	0
504,346	Siphoviridae	Pseudomonas phage PAJU2	BACTERIA	1	0	0	0
578,234	Siphoviridae	Lactobacillus phage Lv-1	BACTERIA	1	0	0	0
1,147,140	Siphoviridae	Salmonella phage SPN3UB	BACTERIA	1	0	0	0
10,724	Siphoviridae	Bacillus phage SPP1	BACTERIA	1	0	0	0
2,560,250	Siphoviridae	Vieuvirus	BACTERIA	1	0	0	0
2,759,464	Siphoviridae	Klebsiella virus KpV2811	BACTERIA	1	0	0	0
446,529	Siphoviridae	Microbacterium phage Min1	BACTERIA	0	1	0	0
482,822	Siphoviridae	Escherichia phage DE3	BACTERIA	0	1	0	0
1,982,251	Siphoviridae	Pahexavirus	BACTERIA	0	0	1	0
1,881,262	Myoviridae	Vibrio phage 1.202.O._10N.222.45.E8	BACTERIA	1	0	0	0
2,588,093	Myoviridae	Pantoea phage vB_PagM_AAM37	BACTERIA	1	0	0	0
10,682	Myoviridae	Enterobacteria phage P7	BACTERIA	0	1	0	0
2,733,124	Myoviridae	Phapecoctavirus	BACTERIA	0	0	1	0
363,555	Myoviridae	Lactobacillus phage KC5a	BACTERIA	0	0	1	0
2,843,161	Schitoviridae	Zicotriavirus	BACTERIA	1	0	0	0
186,846	Salasmaviridae	Salasvirus	BACTERIA	0	0	1	0

## Results of the bacterial Microbiome analysis

**Fig. 1:**
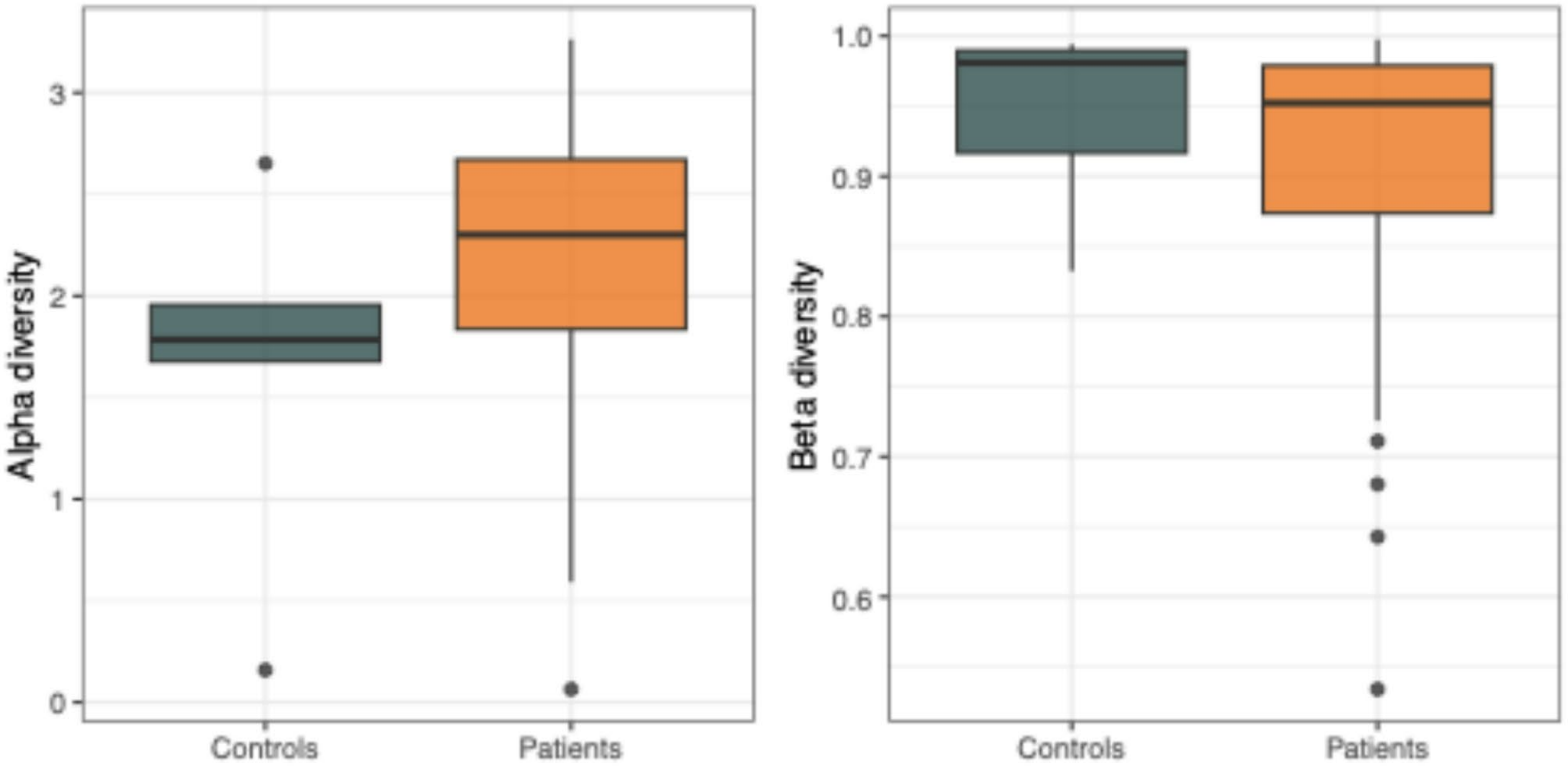
Alpha-diversity calculated by the Shannon method and the beta-diversity by principal coordinate analysis on Bray-Curtis dissimilarity between patients and controls.

The alpha-diversity calculated by the Shannon method (species diversity and abundance) and the beta-diversity by principal coordinate analysis on Bray-Curtis dissimilarity (differences in species composition between two or more habitats) between patients and controls is shown in Fig. 1.

Alpha-diversity was considerably higher in the OAB group, whereas beta-diversity was considerably lower; however, none of these differences reached statistical significance (t-test, *p* = 0.396 and permanova, p.adjusted = 0.183). In Supplementary Fig. 4 we display relative abundances of bacterial genera either within groups of patients and controls, within the four pools used for assessment of viral microbiota, and for each study participant individually.

Thereby, we found considerable differences for a number of microorganisms on the genus level between patients and controls, such as higher levels of *Streptococcus* in patients. However, due to the low sample size, we only observed statistically significant differences for *Veillonella* and *Bacteroides* (Supplementary Fig. 5; t.test, p.adj = 0.00160, respectively). No association could be found between the bacteriophages and bacterial microbiota found in the patients’ samples.

## Discussion

### Key results

We identified a total of 21 different viruses in urethral samples of patients with overactive bladder syndrome, but no valid detections were retained in the control group after analysis. Thus we were able to show a significant difference of the urethral virome between OAB patients and controls. Among the detected viruses in urethral swab samples of OAB patients were both human (eukaryotic viruses) and bacterial viruses (bacteriophages). The most abundant human viruses belong to the family of Papillomaviridae whereas the most abundant bacteriophages belong to the family of Siphoviridae.

Furthermore we detected a considerably higher diversity and abundance of bacteria in OAB samples, with statistically significant differences of *Veillonella* and *Bacteroides*.

### Interpretation

Our findings from viral and bacterial microbiome analysis from urethral swab samples are in accordance with the findings from previous studies, reporting a generally greater diversity and higher abundance of bacteria in OAB samples compared to controls^16,17,19^. This is however the first report of the existence of a viral microbiome in urethral swab samples of OAB patients. It seems interesting that none of the control samples showed no valid viral detections. The detected viruses can be summarized into two main groups – eukaryotic viruses (human papillomavirus, torque teno virus, and Human endogenous retrovirus K113) and bacteriophages.

### Eukaryotic viruses in urethral swab samples of patients

In our samples, the most abundant human viruses were papillomaviridae (human papillomavirus type 56 and type 87). Human papillomavirus (HPV) is a highly specific virus, which only infects humans and of which more than 200 different types exist^44^. Certain types of HPV are well-known for their role in the pathogenesis of epithelial cancers, specifically for cervical cancer in females^44^. The point-prevalence of HPV in pre-vaccination populations is estimated to 10% of women globally^45^, and around 20% in Austria^46^. HPV 56 is considered a high-risk type for the development of cervical cancer^47^, whereas HPV 87 is not specifically associated with any cancer type. It has been previously speculated that a larger diversity of genital microbiota with reduced *Lactobacillus* spp., as also seen in OAB patients^16,17,19^, may contribute to HPV infection in women^48^. In the present study however, the occurrence of lactobacilli was comparable between patients and controls. Thus, in 8 out of 15 patients and 3 out of 5 controls, lactobacilli were absent or their relative abundance did not exceed 5% (Supplementary Fig. 4). Furthermore, HPV is known to be more persistent in immunocompromised individuals. A study found that postmenopausal women with high-grade squamous intraepithelial lesions (HSIL) associated with HPV had significantly more overactive bladder (OAB) symptoms than those with low-grade lesions (LSIL). The authors suggest that HPV-induced inflammation may play a role in the development of OAB, particularly in immunocompromised individuals^49^. Whether OAB patients are per se immunocompromised and thus more vulnerable for HPV infections and microbial shift, or if an HPV infection can trigger OAB symptoms is yet to be determined by further investigations.

Torque Teno Virus (TTV 19 and 29) is a ubiquitous and highly prevalent virus, which has previously been described as a marker of immunocompetence or “surrogate marker of immune status”^50^. Available data suggests that TTV replicates strongly in a setting of immunosuppression in humans^50,51^, thus TTV viral load is discussed as an indicator of immunosuppression. Human endogenous retroviruses are a family of viruses within our human genome, integrated 40 million years ago and thus inherited by successive generations, which constitute about 1% of our genome^52,53^. Of these, HERV-K is the only one which produces viral particles and evidence suggests that HERV-K 113 is the youngest sequence^53^. HERV-K113 shows a wide-range of prevalence rates depending on ethnicity and geographical location, with low prevalence rates ranging from 0% to 21,8%. It has previously been discussed as genetic risk factor for some types of autoimmunity^54^.

Bacteriophages from the Siphoviridae, Myoviridae, Schitoviridae and Salasmaviridae family were the most abundant bacterial viruses detected in our OAB samples, including phages of Pseudomonas, Salmonella, Klebsiella, Escherichia and Enterobacteria. None of these bacteria constitute the healthy urogenital microbiome. Bacteriophages are generally known for their capacity to directly shape the bacterial microbiome of a human body site. Thus, the absence of *Escherichia* in the patient pool 2 could be due to the presence of Escherichia phage DE3, and the lower abundance of lactobacilli in patient pool 3 could be due to Lactobacillus phage KC5a (Table 2, Supplementary Fig. 4).

In addition to the viral urethral microbiome, which we report for the first time, our bacterial microbiome analysis revealed significantly elevated levels of *Veillonella* and *Bacteroides* in samples from individuals with OAB.

*Veillonella* is a prototypical oral microbe that has previously been linked to gastrointestinal diseases and chronic inflammation. Whilst this bacterium is normally a poor gut colonizer and does not cause disease in healthy subjects, previous research could demonstrate that gut injury and inflammation provides favorable conditions for the growth and expansion of *Veillonella*^55^. Nothing is known however on the role of *Veillonella* in the healthy urogenital tract. Based on our data we can suggest and once more postulate a state of chronic inflammation in OAB, which would then provide the environment for the expansion of *Veillonella*. *Bacteroides*, on the other hand, take a role as beneficial organisms, but likewise as potentially opportunistic pathogens –based on their locations in the host. Common sites of *Bacteroides* infection and disease conditions range from brain abscess to pneumonia, endocarditis and inflammatory bowel disease^56^. *Bacteroides* have been linked to urinary tract infections, but no further knowledge exists on their status within the urogenital tract or in OAB patients.

### Limitations

The study has several limitations, including its small sample size, which may limit the generalizability of the results to a larger population of OAB patients. Additionally, pooling samples to enhance viral detection reduces individual-level data, but this approach was necessary due to the low viral load in urethral swabs. The study primarily focused on detecting viral presence and comparing groups, rather than examining individual variation. Future research will include a larger sample size with individual samples, allowing for a more detailed analysis. Results need to be replicated in larger sample sizes, possibly with subgroup analyses to further differentiate between OAB types (e.g. wet versus dry; influence of medication intake, etc.) and to account for potential confounders such as hormone therapy, comorbidities, and medication use.

## Conclusions

Results of this study suggest a difference in the urethral virome between women with OAB and healthy controls, as we could detect a variety of viruses in OAB samples, but no valid viral detections I in the controls. This is in line with the previously reported higher diversity of the bacterial microbiome in urine samples of women with OAB compared to controls. When looking deeper into the detected virus families and species, we might postulate a unique microbial pattern of female OAB patients. This pattern suggests an interplay of immunosuppression, autoimmune processes and a larger diversity of bacterial and viral microbes. Results from this study also confirm our previous proteomics data of OAB urine samples, showing a larger diversity of proteins involved in immunological pathways, suggesting a state of chronic cellular stress and loss of protective factors in OAB^9^.

These findings suggest that microbiome restoration strategies may offer novel therapeutic avenues for patients with OAB, potentially improving both symptom burden and quality of life.

## Electronic supplementary material

Below is the link to the electronic supplementary material.

Supplementary Material 1

## Data Availability

All data supporting the findings of this study are available within the paper or under supplementary materials. The authors confirm that the data supporting the sequencing findings in this study are available at NCBI (Bio Project ID: PRJNA1156392).
