# Supplementary material for: Women suffering from overactive bladder syndrome exhibit a higher urethral viral abundance compared to healthy controls: a pilot study
Source: Sci Rep. 2025 Jun 3;15:19484. doi: 10.1038/s41598-025-98780-9 (PMC12134358; doi:10.1038/s41598-025-98780-9)
Supplement: Supplementary file 1 — Supplementary Material 1 [file 41598_2025_98780_MOESM1_ESM.docx]

Supplementary materials

**Supplementary Table 1:** Processing of viral microbiome raw data.

| **Pool ID** | **Raw read pairs, n** | **Filtered read pairs, n** | **% aligned to human** | **% aligned to neg** |
| --- | --- | --- | --- | --- |
| 1 | 3,298,573 | 3,272,230 | 48.39 | 4.15 |
| 2 | 782,694 | 777,736 | 57.27 | 0.45 |
| 3 | 989,412 | 891,888 | 54.96 | 0.22 |
| 4 | 524,126 | 520,690 | 51.18 | 0.29 |

**Supplementary Table 2**: Contig assembly with SPADES

| **Pool ID** | **Total contigs** | **Total bp** | **Min bp** | **Max bp** | **Deduplicated** |
| --- | --- | --- | --- | --- | --- |
| 1 (sub) | 16,176 | 2,266,977 | 55 | 1,641 | 10,901 |
| 2 | 21,002 | 2,572,511 | 55 | 1,858 | 12,529 |
| 3 | 20,944 | 2,822,584 | 55 | 1,487 | 13,997 |
| 4 | 13,770 | 2,120,295 | 55 | 4,254 | 10,514 |

**Supplementary Table 3:** Contig information.

| **Pool ID** | **Total contigs** | **Min bp** | **Max bp** |
| --- | --- | --- | --- |
| 1 (sub) | 4237 | 200 | 1,641 |
| 2 | 3688 | 200 | 1,858 |
| 3 | 4737 | 200 | 1,487 |
| 4 | 4049 | 200 | 4,254 |

**Supplementary Table 4:** Blastn results for predicted BeAn58058 contigs.

| **Pool/Viral contig** | **Percent Identity** | **Coverage** | **Blastn** | **Accession number** |
| --- | --- | --- | --- | --- |
| 1/152 | 92% | 97% | homo sapiens | AC023509.42 |
| 1/216 | 92% | 59% | homo sapiens | NG_042269.1 |
| 1/239 | 99% | 100% | homo sapiens | CP068257.2 |
| 2/58 | 99% | 96% | homo sapiens | FJ515846.1 |
| 2/189 | 98% | 99% | homo sapiens | AC278001.1 |
| 2/406 | 100% | 100% | homo sapiens | CP068259.2 |
| 3/47 | 100% | 100% | homo sapiens | AC004905.2 |
| 3/51 | 98% | 79% | homo sapiens | CP068256.2 |
| 3/171 | 100% | 100% | homo sapiens | FO704660.2 |
| 4/195 | 100% | 100% | homo sapiens | CP034500.1 |
| 4/268 | 98% | 100% | homo sapiens | AP023482.1 |
| 4/791 | 96% | 87% | homo sapiens | AP023479.1 |
|  |  |  |  |  |

**Supplementary Table 5.** Blastv results for different predicted BeAn58058 contigs.

| **Pool/Viral contig** | **Percent Identity** | **Coverage** | **Blastv** | **Accession number** |
| --- | --- | --- | --- | --- |
| 1/152 | 91% | 65% | BeAn58058 | NC_032111 |
|  | 88% | 92% | HIV | MZ766785 |
| 1/216 | 86% | 66% | BeAn58058 | NC_032111 |
|  | 84% | 66% | HIV | MT154980 |
| 1/239 | 84% | 28% | HIV | MT154980 |
| 2/58 | 80% | 57% | HIV | MK383886 |
| 2/189 | 94% | 18% | HERV | U27241 |
|  | 92% | 20% | HIV | MK458165 |
| 2/406 | 92% | 26% | HIV | MK458255 |
| 3/47 | 93% | 7% | BeAn58058 | NC_032111 |
|  | 100% | 5% | HIV | MK384275 |
| 3/51 | 90% | 51% | BeAn58058 | NC_032111 |
|  | 95% | 10% | HIV | MK384356 |
| 3/171 | 90% | 26% | BeAn58058 | NC_032111 |
|  | 92% | 54% | HERV H | AJ289710 |
| 4/195 | 82% | 55% | BeAn58058 | NC_032111 |
|  | 92% | 10% | HIV | OM207634 |
| 4/268 | 88% | 30% | BeAn58058 | NC_032111 |
|  | 86% | 80% | HIV | MT154980 |
| 4/791 | 86% | 83% | BeAn58058 | NC_032111 |
|  | 92% | 84% | HIV | MT154980 |

**Supplementary Figure 1:** Alignment of predicted BeAn58058 contigs to the reference genome of BeAn 58058 and HIV showing a conserved region of ~190bp.


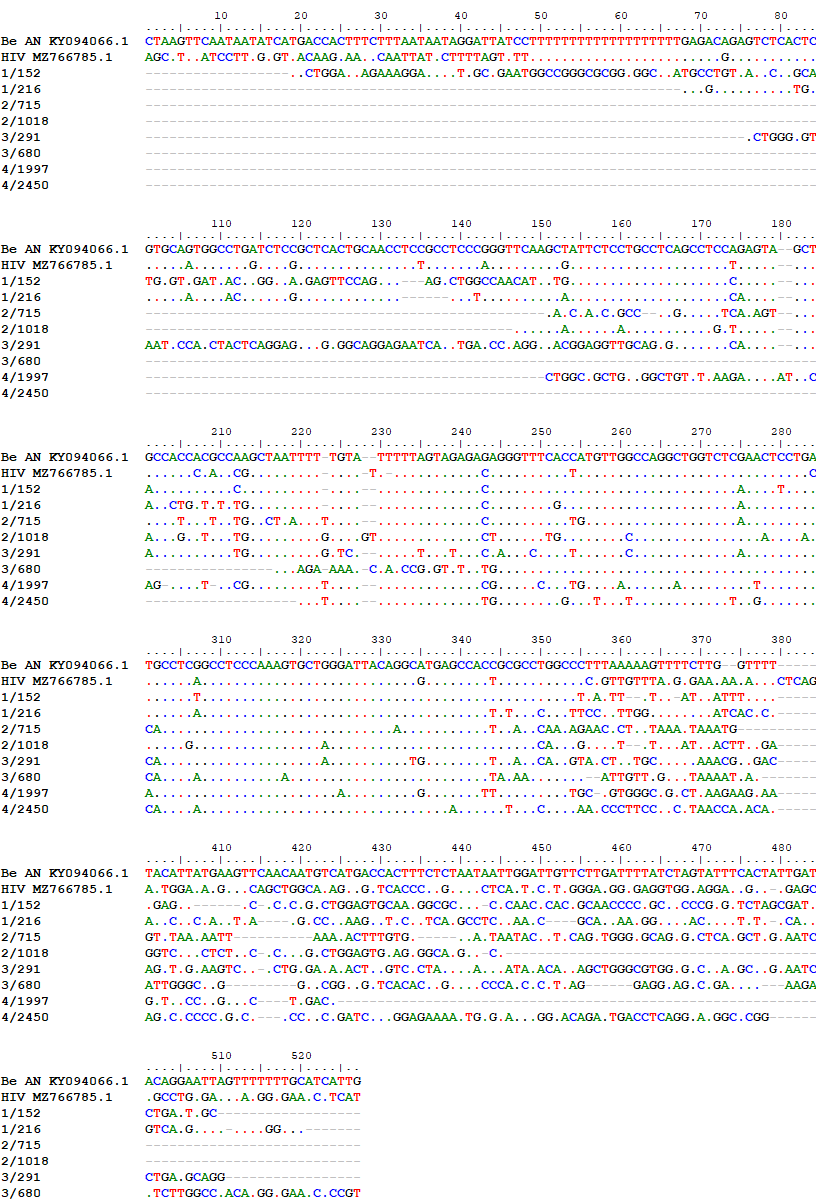


**Supplementary Table 6.** Blastn results for relevant detected viruses with Kaiju.

| **Spades prediction** | **Pool/Contig ID** | **Percent Identity** | **Coverage** | **Blastn** | **Accessionnumber** |
| --- | --- | --- | --- | --- | --- |
| Alphapapillomavirus 3 | 1/1429 | 99% | 100% | Human papillomavirus 87 | KU298942.1 |
| Alphapapillomavirus 3 | 1/1658 | 100% | 100% | Human papillomavirus 87 | KU298942.1 |
| Human papillomavirus type 53 | 1/1250 | 100% | 100% | Human papillomavirus 56 | LR861980.1 |
| Macaca mulatta papillomavirus 6 | 1/1945 | 99.6% | 100% | Human papillomavirus 87 | OP712082.1 |
| Torque Teno Virus 19 | 1/2676 | 98% | 100% | Torque teno virus AZ7_4 | MW679006.1 |
| Torque Teno Virus 29 | 2/1151 | 92% | 100% | Anelloviridae sp. isolate SPI9 | MZ825016.1 |
| Human endogenous retrovirus K113 | 1/1439 | 100% | 100% | Endogenous virus HERV-K | JN675090.1 |
| Human endogenous retrovirus K113 | 3/3147 | 100% | 86% | Endogenous virus HERV-K | JN675026.1 |

**Supplementary Figure 2:** Maximum Likelihood tree assembled using the Contig 1250 (Human papillomavirus type 53) from pool 1 and the reference genomes of HPV types of the same family Alphapapillomavirus 6. As an outgroup HPV 59 of the family Alphapapillomavirus 7 was used.


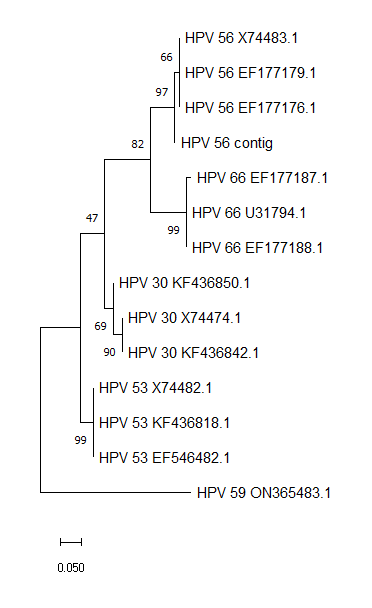


**Supplementary Figure 3:** Maximum Likelihood tree shows the consensus of the three contigs (Alphapapillomavirus 3 and Macaca mulata papillomavirus 6 with SPADES assembly) and the reference genomes of HPV types of the same family Alphapapillomavirus 3. As an outgroup HPV 2 of the family Alphapapillomavirus 4 was used.


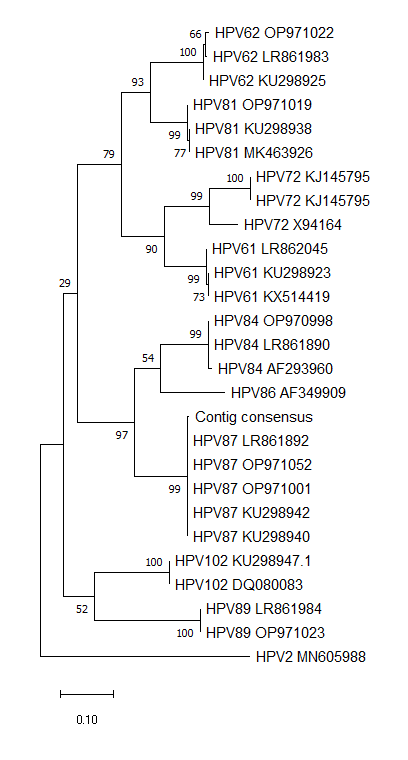


**Supplementary Figure 4:** Relative abundances of bacterial genera either within groups of patients and controls (A), within the four pools used for assessment of viral microbiota (B), and for each study participant individually (C)

**Supplementary Figure 5:** Statistically significantly higher relative abundance of the genera *Veillonella* and *Bacteroides* in patients in comparison to controls

**
